# *Biochemical Society Transactions*: celebrating 50 years

**DOI:** 10.1042/BST20230828

**Published:** 2023-08-23

**Authors:** James M. Murphy

**Affiliations:** 1 The Walter and Eliza Hall Institute of Medical Research, 1G Royal Parade, Parkville, Melbourne, Victoria 3052, Australia; 2 Drug Discovery Biology, Monash Institute of Pharmaceutical Sciences, Monash University, Parkville, VIC 3052, Australia

**Keywords:** biochemical society, biochemical society transactions

## Abstract

*Biochemical Society Transactions* is the reviews journal of the Biochemical Society. Publishing concise reviews written by experts in the field it provides a timely snapshot of the latest developments across all areas of the molecular and cellular biosciences. Elevating authors’ ideas and expertise, each review includes a perspectives section where authors offer comment on the latest advances, a glimpse of future challenges and highlighting the importance of associated research areas in far broader contexts. As *Biochemical Society Transactions* celebrates its 50th birthday, the current Editor-in-Chief looks back on the journal's history, and looks forward to the next 50 years.

2023 marks the 50th birthday of *Biochemical Society Transactions* (*BST*). I am honoured to have been appointed as the Editor-in-Chief on such an auspicious occasion. I succeed Professor Colin Bingle, who has overseen the transition of *BST* from a journal whose content was tethered to Biochemical Society conferences to *the reviews journal of the Biochemical Society* where minireviews are sought from far and wide. We are grateful to Colin for his leadership of the journal over the past 8 years, and for leaving the journal in a position of strength.

*BST* has undergone an incredible evolution over the past 50 years. Initially, the journal was a venue to publish abstracts associated with Biochemical Society focused meetings, of which there were around 11 per year, in addition to book reviews of scientific texts, occasional obituaries, and transcripts of award lectures, which included figures and a photograph of the awardee. There is a rich history in the archives, which makes for entertaining reading. Abstracts were often several pages long and typically included a summary figure or table and thorough citation of the background to the scientific problem. Early authors included some of the most prominent figures of the time, including many Nobel Laureates. Max Perutz was published in the first issue in January 1973; Ernst Chain published many abstracts in the area of his focus later in his career — heart metabolism — in 1973 and 1974; César Millstein contributed on cell-free methods to produce antibodies; Edwin Krebs wrote on new phosphorylase kinase substrates, alongside Mildred Cohn, who wrote on paramagnetic probes to study kinases.

For the first 31 years, *BST* was led by Managing Editors: R. Brian Beechey (1973–1976); David C. Watts (1977–1988); Catherine Rice Evans (1989–1992); Keith Snell (1993–2001); and John Wrigglesworth (2002–2004). The first Honorary Editor, David Richardson, was appointed in 2005 and was succeeded by Colin Bingle in 2015. Colin was appointed as the inaugural Editor-in-Chief in 2016. Each editorial transition is marked by innovations. In 1977, minireviews first appeared in *BST* in addition to Communications, which were brief summaries of presentations at Biochemical Society conferences. Minireviews became the currency of the journal at the end of 2002, when abstracts were no longer published. Perhaps Dr Hook and the Medicine Show were prognosticators with their obsession with journal covers in the Billboard hit, ‘The Cover of the “Rolling Stone”', 50 years ago; however, it was only October 2006 when *BST* first published cover art. The range of images over the years is timeless; the images of cells, proteins, network and pathway diagrams are as striking today as they were nearly 20 years ago (Figure 1). Since 2018, *BST* transitioned away from Biochemical Society conferences as the principal source of minireview content. Additionally, in collaboration with the Associate Editor team, Professor Bingle restructured author guidelines to contemporise the review format to mandate a presentation item (minimum one table or figure), inclusion of perspectives on the field, an emphasis on literature from the past 3 years, and a word limit of ∼3000.

**Figure 1.**
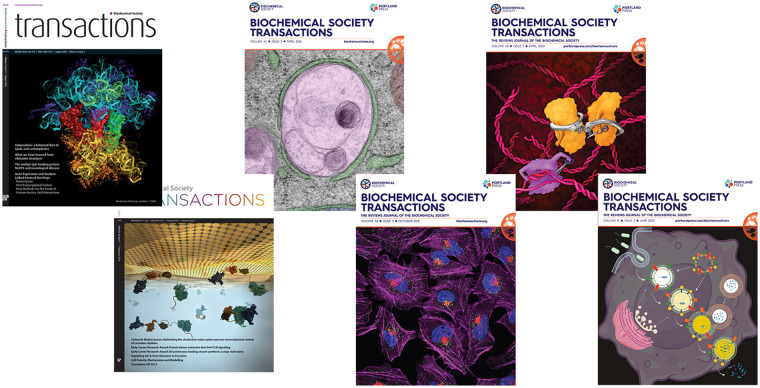
A selection of *Biochemical Society Transactions* cover art.

In 2023, we continue to welcome minireviews from members of the molecular biosciences community. We actively seek reviews from presenters at conferences all over the world and by monitoring for areas of high interest in the field, and are always available to discuss whether your review would be a good fit at *BST*. Our multinational Associate Editor team has expertise across the breadth of biochemistry, ranging from signal transduction and structural biology (James Murphy, Elton Zeqiraj), molecular machines and protein transport in bacteria (Vicki Gold), epigenetics in mammals (Marnie Blewitt) and plants (Jiamu Du), redox biology and cellular damage (Clare Hawkins), microscopy and cellular ultrastructure (Ivan Robert Nabi), systems biology (Johann Rohwer), genome stability and DNA repair in plants (Stefanie Rosa), enzymology and plant biochemistry (Grant Pearce), to mechanotransduction and the extracellular matrix (Alexandre Bruni-Cardoso). Our Associate Editors are practising researchers who seek to minimise the obstacles to members of our community publishing their work. There are no page charges to publish your work in *BST*, and many institutions have signed Read & Publish deals with Portland Press (the publishing arm of the Biochemical Society) to enable you to publish your work under a Gold Open Access licence at no cost to you. We thank all of our contributors for trusting us with publishing their work, we are grateful to our expert reviewers and editorial office staff for helping us maintain high standards in the journal, and we look forward to the next 50 years of innovative publishing at *Biochemical Society Transactions*.

## Competing Interests

The author declares that there are no competing interests associated with this manuscript.

## Open Access

Open access for this article was enabled by the participation of University of Melbourne in an all-inclusive *Read & Publish* agreement with Portland Press and the Biochemical Society under a transformative agreement with CAUL.

